# Loss of miR-200c-3p promotes resistance to radiation therapy via the DNA repair pathway in prostate cancer

**DOI:** 10.1038/s41419-024-07133-3

**Published:** 2024-10-16

**Authors:** Maureen Labbé, Manon Chang, Benjamin Saintpierre, Franck Letourneur, Laurence de Beaurepaire, Joëlle Véziers, Sophie Deshayes, Marine Cotinat, Jean-François Fonteneau, Christophe Blanquart, Vincent Potiron, Stéphane Supiot, Delphine Fradin

**Affiliations:** 1 Nantes Université, Inserm UMR 1307, CNRS UMR 6075, Université d’Angers, CRCI2NA, F-44000 Nantes, France; 2 Université de Paris, Institut Cochin, Inserm, CNRS, 75014 Paris, France; 3 ONIRIS, INRAE, IECM, Nantes, France; 4 ONIRIS, B-FHIT, Nantes, France; 5 Nantes Université, Oniris, CHU Nantes, INSERM, Regenerative Medicine and Skeleton, RMeS, UMR 1229, F-44000 Nantes, France; 6 Institut de Cancérologie de l’Ouest, Saint Herblain, Nantes, France; 7 Nantes Université, CNRS, US2B, UMR 6286, F-44000 Nantes, France

**Keywords:** Radiotherapy, miRNAs, Double-strand DNA breaks

## Abstract

Radiotherapy represents a major curative treatment for prostate cancer (PCa), but some patients will develop radioresistance (RR) and relapse. The underlying mechanisms remain poorly understood, and miRNAs might be key players in the acquisition and maintenance of RR. Through their encapsulation in small extracellular vesicles (EVs), they can also be relevant biomarkers of radiation response. Using next-generation sequencing, we found that miR-200c-3p was downregulated in PCa RR cells and in their small EVs due to a gain of methylation on its promoter during RR acquisition. We next showed that its exogenous overexpression restores the radiosensitivity of RR cells by delaying DNA repair through the targeting of HP1α. Interestingly, we also observed downregulation of miR-200c-3p expression by DNA methylation in radiation-resistant lung and breast cancer cell lines. In summary, our study demonstrates that the downregulation of miR-200c-3p expression in PCa cells and in their small EVs could help distinguish radioresistant from sensitive tumor cells. This miRNA targets HP1α to delay DNA repair and promote cell death.

## Introduction

Prostate cancer (PCa) is the most common cancer in men, and its incidence rate is increasing worldwide [1]. For localized disease, the current treatment modalities are radical prostatectomy, radiation therapy (RT) with or without androgen deprivation therapy (ADT), and active surveillance. Conventional RT is based on daily fractions of 2 Gray (Gy), up to a total dose of 70–80 Gy [2]. The therapeutic response to RT is determined in part by intrinsic radiosensitivity, which is the level of enzymes that repair DNA damage. Indeed, RT induces cell death through a variety of DNA lesions, including base damage, single-strand breaks (SSBs), and DNA double-strand breaks (DSBs). In response to these damages, cells trigger the DNA damage response (DDR), which results in cell cycle arrest to provide the cell time to repair DNA through homologous recombination (HR) or non-homologous end-joining (NHEJ) [3]. Tumor cells with efficient DNA repair capacities could, therefore, be more resistant to radiation. However, many other factors, such as genetic and epigenetic alterations, the tumor itself, or the tumor microenvironment, could also influence the therapeutic efficacy of RT. It is crucial to better understand the underlying mechanisms of radiation resistance to develop new treatments, but also sensitive, robust and minimally invasive tools to predict the response to RT.

microRNAs (miRNAs) are attractive candidate biomarkers. They are easily purified and highly stable in body fluids, especially due to their encapsulation in small extracellular vesicles (small EVs) [4], and their expression can distinguish healthy individuals from cancer patients [5, 6]. Moreover, miRNAs are key posttranscriptional regulators of gene expression. By fine-tuning gene expression, miRNAs are important modulators of DDR, cell death, tumor aggressiveness, and the tumor microenvironment, and, consequently, of the tumor response to RT [7].

Previous studies have described the role of some miRNAs in radiation resistance (RR) in PCa, [8–10], but it is unknown whether these miRNAs can be released and used as biomarkers of the response to RT in patients. To go further in the understanding of the underlying mechanisms of RR in PCa and address this limitation, we used small next-generation sequencing (NGS) to comprehensively identify miRNAs that may be involved in the RR and release by PCa cells. We found that miR-141-5p and miR-200c-3p are strongly downregulated in PCa RR cells. We further demonstrated that only miR-200c-3p expression can be followed outside of tumor cells in their small EVs, indicating that miR-200c-3p could be a valuable noninvasive prognostic biomarker. Functionally, we showed that miR-200c-3p directly targets HP1α, a protein involved in DNA repair processes, which explains its role in RR. These findings help to characterize radiation resistance in PCa and suggest a potential new biomarker of the response to RT.

## Materials and methods

### Cell lines

DU145 human prostate adenocarcinoma cell line; HEK-293 cells, H358, H838, H1437, A549, H1975 human lung cancer adenocarcinoma cell lines, MCF-7 and MDA-MB-231 breast cancer cell lines were purchased from the American Type Culture Collection (ATCC-LGC Standards). The lung cancer cell lines ADCA72, ADCA153, ADCA211, and ADCA233 were established from patient pleural effusion in our laboratory [11]. All patients provided informed consent (DC-2011-1399). PC3 human prostate adenocarcinoma cell line and its RR counterpart were graciously provided by Dr. Stanley K Liu.

Prostate adenocarcinoma cell lines and HEK-293 cells were cultured in DMEM (Gibco, Cat# 21969035). Lung and breast cancer cell lines were cultured in RPMI-1640 (Eurobio, Cat# CM1RPM00-01). The two media were supplemented with 10% fetal bovine serum (Corning, Cat# 35-015-CV), L-glutamine (2 mM, Gibco, Cat# 25030-024), and penicillin–streptomycin (100 µg/mL, Gibco, Cat# 15140-122). The cell lines were tested each week for mycoplasma contamination using a PlasmoTest Mycoplasma detection kit (InvivoGen, Cat# Rep-ptrk) according to the manufacturer’s protocol; contaminated cells were discarded.

### Primary healthy cells

Primary human umbilical vein endothelial cells were isolated in the laboratory as previously described [12] from three human umbilical cords (Nantes CHU bio-collection number DC-2017-2987) and cultured in fully supplemented EGM2 medium (Merck, Sigma-Aldrich, Cat# C-22011).

Melanocytes (ATTC, Cat# PCS-200-013) were purchased from ATCC and cultured in dermal cell basal medium (ATCC, Cat# PCS-200-030) supplemented with one adult melanocyte growth kit (ATCC, Cat# PCS-200-042). The culture medium was changed every 48 hours and kept in culture for 6 weeks.

Peripheral blood mononuclear cells were obtained from healthy donors (ethics agreement CPDL-PLER-2018 021), and CD8+ fraction was immediately isolated using EasySep Human CD8 + T cell Enrichment Kit (Stemcell, Cat# 19159) according to the manufacturer’s protocol. They were grown in RPMI-1640 (Eurobio, Cat# CM1RPM00-01) supplemented with 8% human serum (local production), L-glutamine (2 mM, Gibco, Cat# 25030-024) and penicillin-streptomycin (100 µg/mL, Gibco, Cat# 15140-122).

All cells were cultivated at 37 °C in a 5% CO2 atmosphere.

### Generation of radiation-resistant cells

Radioresistant cells were generated by treatment with mock irradiation (DU145 cells) or ionizing radiation (DU145 RR cells) delivered using a 160 kV, 6.3 mA Faxitron X-ray irradiator (Faxitron, Tucson, AZ, USA) for a daily 2 Gy dose administered 5 days per week, with this process repeated for a total of 90 Gy. A DU145 60 Gy cell line was established from cells that had undergone a total of 60 Gy of irradiation. The irradiation scheme was done according to the conventional patients.

### Radiation clonogenic survival assay

Cells were plated in triplicate in six-well plates. Radiation was performed using a Faxitron X-ray irradiator (Faxitron, Tucson, AZ, USA). The radiation regimens were as follows: 160 kV, 6.3 mA, for 1.2, 2.4, 3.7 min for radiation doses of 2, 4, and 6 Gy, respectively. For all clonogenic assays, the plates were incubated for 10–14 days to allow for colony formation and then stained with a crystal violet staining solution. The number of colonies was counted (defined as >50 cells), and the surviving fraction of irradiated cells was calculated relative to the plating efficiency of mock-irradiated cells. The area under the curve (AUC) representing the mean inactivation dose (MID) was calculated using GraphPad Prism. The radiation protection factor (RPF) was calculated by dividing the MID of the test cells by the MID of the control cells.

### Small EVs purification

Cancer cell line-derived small EVs were isolated from cancer cells at 90% confluence. The cells were cultured in media supplemented with 10% exosome-depleted fetal bovine serum (Gibco, Cat# A2720801) for 24 h as previously described [13]. Briefly, differential centrifugations were performed to isolate small EVs: 5 min at 300 g, 10 min at 2000 × *g*, 30 min at 10,000 × *g*, and two runs of 120 min at 100,000 × *g*. The final pellet containing small EVs was resuspended in PBS. The Beckman Optima L-80XP instrument with a SW32Ti rotor was used to ultracentrifuge small EVs from cell lines.

### Small EVs quantification

The size and concentration distribution of small EVs were quantified with tunable resistive pulse sensing (TRPS) technology with qNano instrument (IZON). All samples were diluted in PBS with 0.03% Tween 20 and recorded at two different pressures on a NP150 nanopore. To calibrate the size and concentration, 110 nm (mode) carboxylated polystyrene beads (IZON) were used. For each recording, at least 500 particles were counted for a minimum rate of 100 particles/minute. A stretch between 45 and 47 with a voltage between 0.5 and 0.7 V was used to achieve a stable current between 135 and 145 nA.

### Western blot

Small EVs and cells were lysed with RIPA buffer (Thermo Scientific, Cat# 89900) containing Proteinase Inhibitor Cocktail (Sigma-Aldrich, Cat# 4693124001). To study proteins located in the nucleus (HP1α), cells were lysed with NE-PER Nuclear and Cytoplasmic Extraction reagents (Thermo Scientific, Cat# 78833) according to the manufacturer’s protocol. The protein concentration was determined by BCA (Interchim, Cat# UP40840A). The proteins were denatured at 95 °C for 5 min in Laemmli buffer (Bio-Rad, Cat# 1610747). Then, they were electrophoresed by SDS-PAGE and transferred onto a PVDF membrane. The membrane was blocked for 2 hours at room temperature with 5% non-fat milk or BSA. The membrane was incubated with primary antibody mouse anti-CD63 (Invitrogen Life Technologies, Cat# 10628D) (1 µg/mL), or mouse anti-CD81 (Invitrogen Life Technologies, Cat# 10630D) (1 µg/mL), or mouse anti-TSG101 (Invitrogen, Cat# MA1-23296) (2 µg/mL), or mouse anti-calnexin (Invitrogen Life Technologies, Cat# MA3-027) (4 µg/mL), or mouse anti-H3 (Active Motif, Cat# 39763) (0.5 µg/mL), or rabbit anti-HP1α (Cell Signaling Technology, Cat# 2616) (1:1000) overnight at 4 °C, followed by incubation with a secondary antibody ((goat anti-mouse IgG, HRP-conjugated, Interchim, Cat# 115-036-072) (0.8 µg/mL), or (goat anti-mouse IgG, Alexa Fluor 488, Invitrogen, Cat# A11029) (2 µg/mL), or goat anti-rabbit IgG, HRP, Interchim, Cat# 111-035-006 (0.8 µg/mL)) for another 1 hour. Blots were developed with enhanced chemiluminescent (ECL) substrate (Clarity Western ECL substrate, Bio-Rad, Cat# 170-5061), and protein expression was assessed using a ChemiDocMP Imaging System (Bio-Rad).

### Scanning transmission electron microscopy

Small EVs were investigated by negative stain electron microscopy. Briefly, small EVs were incubated for 20 minutes on formvar-carbon-coated copper 200 mesh grids (AGS162 Agar Scientific, UK). EVs were then washed with PBS and fixed with 1% glutaraldehyde (Sigma-Aldrich, Cat# G5882) dissolved in PBS for 5 minutes. After eight washes with deionized water, the samples were stained with Uranyless (Delta Microscopie, France) for 1 minute. The grids were coated with a thin layer of platinum (0.8 nm) using a Leica EM ACE600 high vacuum sputter coater. Grids with negatively stained vesicles were observed with a GeminiSEM 300 Zeiss scanning electron microscope, equipped with a STEM detector. Observations were made at 29 keV, with a 7.5 µm diaphragm and a working distance of 4 mm.

### RNA isolation

Total RNA (including miRNA and mRNA) was extracted from small EVs derived from tumor cells and from cells with Qiazol reagent (Qiagen, Cat# 217084) and the miRNeasy Mini kit (Qiagen, Cat# 217004) according to the manufacturer’s protocols. Total RNA was eluted in 30 µL of RNAse-free water. The RNA purity and concentration were screened with a Nanodrop 1000 spectrophotometer (Thermo-Fisher Scientific). An Agilent 2100 Bioanalyzer (Agilent) for total RNA (RNA nanochips, Cat# 5067-1511) and for small RNA (small RNA chips, Agilent, Cat# 5067-1548) were used to assess the RNA profiles.

### RNA sequencing and analysis

Small RNA libraries were prepared following the Qiaseq miRNA library prep kit protocol (Qiagen, #331502), starting from 100 ng of the QC-controlled small RNA fraction. Libraries were subsequently sequenced after QC control on a NextSeq 500 (Illumina) using a single Read 75 bp mode. A minimum of 12 million reads were obtained for each sample. The Fastq files were uploaded to the QIAGEN Online Data Analysis Center to align the sequences to miRBase release 21, and the UMI was used to prevent PCR bias. Once each sample was associated with the right group, we performed a standard DESeq2 normalization method with the DESeq2 Bioconductor package [14]. Following the package recommendations, we used the Wald test with the contrast function and the Benjamini-Hochberg FDR control procedure to identify the differentially expressed miRNAs.

3′Sequencing RNA profiling was performed by the GenoBird Platform using a NovaSeq 6000 (Illumina). The raw sequence reads were filtered based on quality using FastQC. Adapter sequences were trimmed off the raw sequence reads using Cutadapt. The reads were subsequently aligned to the reference genome using BWA. Differential expression analysis was performed with the DESeq2 Bioconductor package [14]. Following the package recommendations, we used the Wald test with the contrast function and the Benjamini-Hochberg FRD control procedure to identify the differentially expressed RNA. Additionally, we identified the KEGG (Kyoto Encyclopedia of Genes and Genomes) pathways associated with the differentially expressed RNAs, using PathfindR [15].

### Prediction of the mRNA targets of miR-200c-3p

The miR-200c-3p target mRNAs were predicted with multimiR R packages [16]. Filtration of the predicted miRNA–target interaction database was performed with Diana, TargetScan, miRanda, and miRDB.

### Real-time quantitative PCR

miRNA expression in PCa small EVs and cells was analyzed by Taqman. miRNAs were reverse transcribed with the TaqMan Advanced miRNA cDNA synthesis Mix (Life Technologies, Cat# A28007), and quantified on a QuantStudio3 system (Applied) using the Taqman Fast Advanced Master Mix (Life Technologies, Cat# 4444963) and primers (Life Technologies). Each reaction sample was run in duplicate. Using our NGS data, we identified hsa-miR-3135b as an internal reference gene for PCa small EVs. The sequence of the primers used is available in Supp Table 1.

miRNAs expressions in PCa cells, lung cancer cells, and breast cancer cells were analyzed via a miRCURY LNA assay. miRNAs were reverse transcribed with a miRCURY LNA RT kit (Qiagen, Cat# 339340) according to the manufacturer’s protocol. Quantitative PCR was performed on a QuantStudio3 system (Applied) using the miRCURY LNA SYBR Green PCR kit (Qiagen, Cat# 339346) and primers. Each reaction sample was run in duplicate. Small nucleolar RNA 44 (snord44) (Qiagen, Cat# 339306 - YP00203902) was used as an internal reference gene for the analysis of miRNA expression in cells. Melting curve analysis was performed at the end of the run to ensure specificity in the amplification. The 2^−ΔΔCt^ method was used to calculate relative changes in expression. The sequences of the primers used are listed in Supp Table 1.

RNAs were reverse transcribed using RevertAid H Minus Reverse Transcriptase (Thermo Scientific, Cat# EP0451), and the RT product was subjected to expression analysis using Maxima SYBR Green/ROX qPCR Master Mix (Thermo Scientific, Cat# K0222). *RPLP0* (Ribosomal Protein Lateral Stalk Subunit P0) gene was used as a reference gene (Supp Table 1). The 2^−ΔΔCt^ method was used to calculate relative changes in expression. Each reaction sample was run in duplicate. Melting curve analysis was performed with a temperature gradient to avoid any issue of nonspecific amplification.

### Methyl-qPCR

Total DNA from cells or small EVs was subjected to digestion at 37 °C for 4 h in 1X CutSmart buffer (Biolabs, Cat# B6004) supplemented with either no enzyme, 100 units of HpaII (Biolabs, Cat# R0171S) or 100 units of MspI (Biolabs, Cat# R0106S). The *MIR200C* promoter was subsequently quantified *via* real-time PCR, as previously described, using specific primers (Supp Table 1). The methylation level was calculated as 100/(1 + E)^Cq2-Cq1^ where E is the PCR efficiency value, Cq2 is the threshold cycle for the digested HpaII sample, and Cq1 is the threshold for the undigested DNA sample.

### Transfection of miRNA mimics

A 66 nM final hsa-miR-200c-3p mimic (Ambion, Cat# 4464066) was transfected into DU145 RR, ADCA72, and A549 tumor cells that had reached 80% confluence using Attractene reagent (Qiagen, #301005). A mimic negative control (Ambion, Cat# 4464058) was also transfected. For the lung cancer cell line A549, a dose-response was also performed. After 24 h of transfection, all the experiments were conducted (clonogenic assay, cell viability, confocal microscopy).

### pLS CBX5 3′UTR plasmid construction and mutation

Putative miR-200c-3p binding sites on the 3′UTR of CBX5 mRNA were predicted using RNAhybrid and IntaRNA [17, 18]. Fragments of 286 nt (position 7508–7918 in 3′UTR *CBX5*), containing potential target sites were synthesized by the company GeneCust (France). The PCR fragment was cloned and inserted into the RenSP luciferase reporter plasmid pLightSwitch 3′UTR (Active Motif, Cat# 32022) by using a NEBuilder HiFI DNA Assembly Cloning kit (NEB, Cat# E5520S). Plasmid construction was validated by DNA sequencing.

pLS 3′UTR *CBX5* mutants were generated by PCR using PrimeStar Max DNA Polymerase (Takara, Cat# R045A). The primers used are described in Supp Table 1. After the initial step at 95 °C for 8 min, 18 cycles were run for 20 s at 95 °C and 15 s at 55 °C followed by 22 s at 72 °C. The PCR mixture was incubated for 1 h at 37 °C with *DpnI* (NEB, Cat# R0176S) to digest the template plasmid DNA. Subsequently, *DpnI* was inactivated by incubating the mixture at 80 °C for 20 min. Mutations were confirmed by DNA sequencing using the primers listed in Supp Table 1.

### Luciferase assay

Twenty nanograms of the RenSP luciferase reporter construct containing a fragment of the 3′UTR of *CBX5*, mutated or not, were transfected into 10,000 HEK-293 cells with miRNA mimics (66 nM final) using Attractene reagent (Qiagen, Cat# 301005). Cell extracts were prepared 24 h after transfection, and luciferase activity was measured using a LightSwitch Luciferase Assay kit (Active Motif, Cat# 32031). Luminescence was measured using a Mithras microplate reader. The data are expressed relative to the data of the cells transfected with the pLS 3′UTR *CBX5* and miR-neg. Technical triplicates were performed for each experiment.

### Cell viability

Five thousand DU145 RR cells were plated in a 96-well plate, and were transfected and incubated overnight before being irradiated at 6 Gy with Faxitron X-ray irradiator (Faxitron, Tucson, AZ, USA). Then, twenty-four hours later, cell viability was assessed using a CellTiter-Glo® Luminescent Cell Viability Assay according to the manufacturer’s protocol (Promega, Cat# G7570). Briefly, cells were incubated for 10 min at a 1:1 dilution with CellTiter-Glo® Reagent. Luminescence was measured using a Mithras microplate reader. The number of living cells relies on cell lysis and the generation of a luminescent signal proportional to the amount of ATP. Technical triplicates were performed for each experiment.

### Confocal microscopy

DU145 RR cells were grown on EZ slides (Millipore, Merck, Cat# PEZGS0816) and transfected with miRNA mimics (66 nM final concentration). After 24 h of transfection, the cells were irradiated at mock-treated (0 Gy) or 6 Gy-treated (3.7 min) with a Faxitron irradiator (Faxitron). After 0 h, 30 min, 6 h or 24 h, cells were fixed in 4% paraformaldehyde for 20 min at RT, permeabilized with Triton-0.75% for 2 h at RT, and then incubated with primary antibodies mouse monoclonal anti-H2AX^Ser139^ (Millipore, Cat# 05-636) (1 µg/mL), primary rabbit polyclonal anti-53BP1 (Abcam, Cat# ab36823) (0.4 µg/mL) diluted in PBS-BSA 2% overnight at 4 °C or 1 h at RT. Then, secondary Alexa antibodies (Goat anti-mouse Alexa 488, Invitrogen, Cat# A11029) (2 µg/mL) and (Goat anti-rabbit Alexa 647, Invitrogen, Cat# A21245) (2 µg/mL) for 1 hour were incubated. The nuclei were then labeled with Hoechst-33342 (Sigma, Cat# 14533) (5 µg/mL). The cells were finally mounted with Prolong (Invitrogen, Cat# P36930) and observed under a Nikon A1 confocal microscope equipped with a 60x/1.4 oil immersion objective (Nikon Instruments). The number of γH2AX and 53BP1 foci per cell was determined using ImageJ. For each condition, more than 50 cells were analyzed.

### Pyrosequencing-based bisulfite PCR analysis

Sixteen CpGs located over 300-bp distance on the promoter *MIR200C* were investigated. CpGs are denominated according to their position in the miRNA sequence. DNA was extracted from cell lines using a Gentra Puregene blood kit (Qiagen, Cat# 1042606). Genomic DNA (500 ng) was treated with EZ DNA methylation-Gold kit according to the manufacturer’s protocol (Zymo Research, Cat# D5005). PCR amplification of the bisulfite-treated DNA was performed using MethylTaq DNA polymerase (Diagenode, Cat# C09010010) and primers (Supp Table 1). A pyrosequencing assay was designed using MethPrimer [19]. Biotinylated single-stranded amplicons were isolated according to the manufacturer’s protocol using the Qiagen Pyromark Q24 Workstation (Qiagen). Pyrosequencing was subsequently performed on a Pyromark Q24 Instrument (Qiagen) with 0.3 µM of each sequencing primer (Supp Table 1). The percentage of methylation for each of the CpGs within the target sequence was calculated using the PyroQ CpG software (Qiagen). CpG methylated human genomic DNA (Thermo Scientific, Cat# SD1131) was used as a methylated control in the assay.

### Statistical analysis

The data are presented as the mean ± SEM of biological replicates. The number of biological replicates of the respective experiment is indicated as n in the figure legends. The statistical details are displayed in the figure legends. The means of two groups were compared using the non-parametric Wilcoxon test. The means of more than two groups were compared with Kruskal–Wallis test. Correlations were assessed by use of Pearson’s correlation coefficient. ns, non-significant, *p < 0.05, **p < 0.001, ***p < 0.001. All the statistical analyses were conducted using R 2022.07.1, with the exception of the clonogenic assays (GraphPad Prism).

## Results

### miR-200c-3p is downregulated in radioresistant cells, and their small EVs

**Fig. 1:**
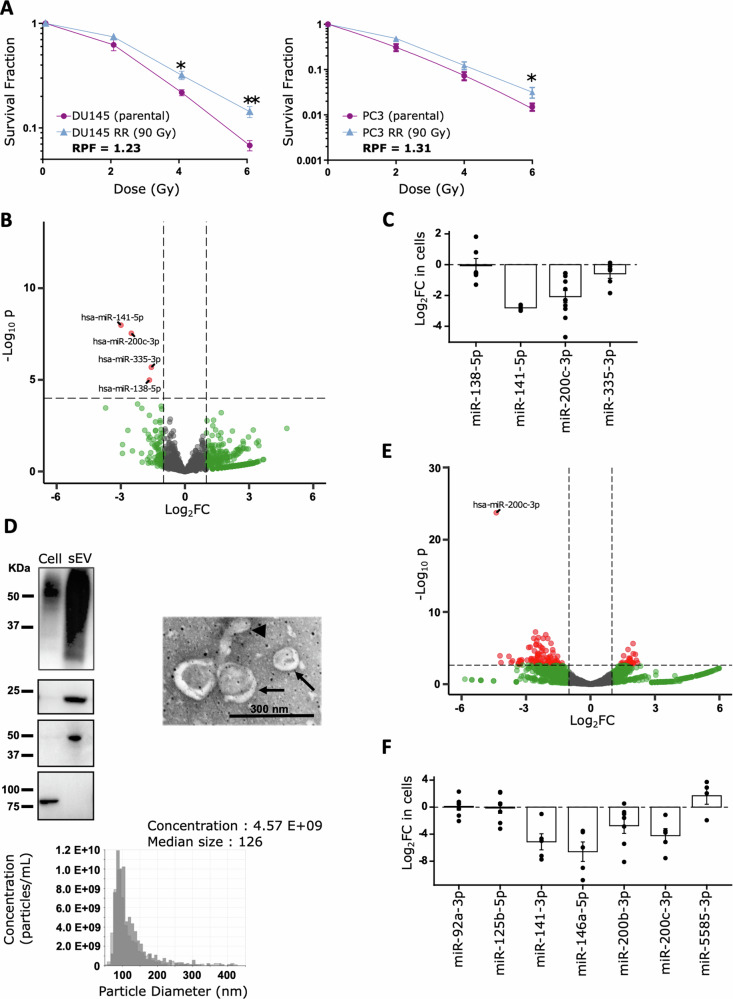
miR-200c-3p downregulation is associated with radioresistance. **A** Radiosensitivity of established radiation-resistant and parental cells was evaluated by colony formation assay. RPF: Radiation Protection Factor. Means and SEM are represented. Wilcoxon test, one side: *p < 0.05, **p < 0.01. *n* = 6 independent experiments. **B** Volcano plot showing the pairwise comparisons of the differential expression of miRNAs in DU145 and PC3 sensitive and RR cells. The red dots on the left represent miRNAs that were significantly downregulated in the radioresistant cells compared to the parental cells. In green, miRNAs with a log2FC > 1 or <−1 are indicated. In red, miRNAs with an adjusted *p* value < 0.05. **C** Log2 Fold Change of miRNAs expressions in DU145 and PC3 cells compared to DU145 RR and PC3 RR cells measured by RT-qPCR (*n* > 4). **D** Characterization of the small EVs released by prostate cell lines: Western blot analysis (3 µg of sample was loaded per well. CD63, CD81, and TSG101 proteins are exosomes markers, and calnexin is a cytosolic protein), scanning electron microscopy (SEM) and TRPS technology. In the electron microscopy image, the arrows indicate intact vesicles, while the arrowhead shows amorphous material compatible with protein aggregates. In the TRPS plot, each replicate is represented by a gray level. **E** Volcano plot of differentially expressed miRNAs in small EVs from DU145 and DU145 RR cells. The red dots on the left represent significantly downregulated miRNAs in small EVs from the DU145 RR cells, and on the right, the upregulated miRNAs compared to small EVs from the DU145 cells. In green, miRNAs with a log2FC > 1 or <−1 are indicated. In red, miRNAs with an adjusted *p* value < 0.05. **F** Log2 Fold Change of miRNAs expressions in DU145 RR-derived small EVs compared to DU145-derived small EVs measured by RT-qPCR (*n* > 4).

To identify new biomarkers of radiation resistance, we first investigated the miRNA content of radiation-resistant and radiation-sensitive PCa cell lines. To generate a resistant DU145 PCa cell line, we exposed parental cells to 2 Gy of X-rays daily until a cumulative dose of 90 Gy was reached. The resistant PC3 human prostate adenocarcinoma cell line and its sensitive counterpart were graciously provided by Dr. Stanley K Liu. To assess their radiosensitivity, parental cells (DU145 and PC3) and their derived resistant cells (DU145 RR and PC3 RR) were exposed to increasing doses of 0, 2, 4, and 6 Gy. Clonogenic survival curves (Fig. 1A and Supp Fig. 1), confirmed that RR cells had significant radiation resistance compared to parental cells (AUC DU154 RR 2.665 vs. DU145 2.164, RPF = 1.23), *p* < 0.01; and AUC PC3 RR 1.698 vs. PC3 1.294, RPF = 1.31, *p* < 0.05).

Next, we conducted a small RNA NGS on these cells. Pairwise analysis identified 4 miRNAs (Table 1, Fig. 1B) that were significantly differentially expressed between DU145 and PC3 RR cells and their parental counterparts after correction for multiple testing. hsa-miR-141-5p, hsa-miR-200c-3p, hsa-miR-335-3p, and hsa-miR-138-5p were significantly downregulated in PCa radioresistant cells compared to the sensitive cells. We confirmed these results by RT-qPCR, except for hsa-miR-138-5p (Fig. 1C).

**Table 1:** List of miRNAs significantly differentially expressed between radioresistant and sensitive prostate cancer cell lines.

miRNA	BaseMean	log2FoldChange	lfc SE	*P* value	*P* adj
hsa-miR-141-5p	322.12	2.99	0.52	1.04e-08	2.87e-05
hsa-miR-200c-3p	4640.50	2.50	0.45	2.90e-08	3.99e-05
hsa-miR-335-3p	989.56	1.57	0.331	2.06e-06	0.00189
hsa-miR-138-5p	853.41	1.66	0.38	1.03e-05	0.00712

We then investigated whether it was possible to detect these miRNAs outside the cells to obtain noninvasive biomarkers. Therefore, we cultured DU145 cells in an exosome-depleted medium at 90% confluence. Twenty-four hours later, the supernatants were collected, and the small EVs were purified by ultracentrifugation. EV purity was evaluated by the presence of exosome markers via western blotting (Fig. 1D). Our results show that CD63, CD81, and TSG101 were specifically enriched in our purifications. The absence of calnexin, a cytoplasmic marker, confirmed the absence of cytoplasmic contaminants in our preparations. To check the morphology of the isolated small EVs, we performed scanning transmission electron microscopy (STEM) (Fig. 1D). The purified particles exhibited a circular morphology with an intact membrane based on negative staining. Using TRPS technology, the median diameter of the small EVs was evaluated at 128 nm, which was comparable to that assessed *via* STEM (Fig. 1D). These observations were within the range of typical small EVs.

A small RNA NGS was then conducted on these small EVs (Fig. 1E, Supp Table 2). Interestingly, miR-200c-3p was also found strongly downregulated in small EVs derived from RR cells compared to parental cells, reflecting its downregulation in resistant tumor cell lines. To validate these results, we performed RT-qPCR Taqman on the 7 detectable miRNAs from the top 10 deregulated (Fig. 1F). We confirmed the significant downregulation of 4 out of 6 identified miRNAs including the miR-200c-3p and the up-regulation of hsa-miR-5585-3p, while the read numbers for miR-1343-5p, miR-3180-5p and miR-4707-5p were too low to be investigated by RT-qPCR Taqman or by LNA PCR assay systems.

In conclusion, these results indicate that miR-200c-3p represents an interesting biomarker of RR in PCa.

### The DNA methylation level at the *MIR200C* promoter correlates with RR

**Fig. 2:**
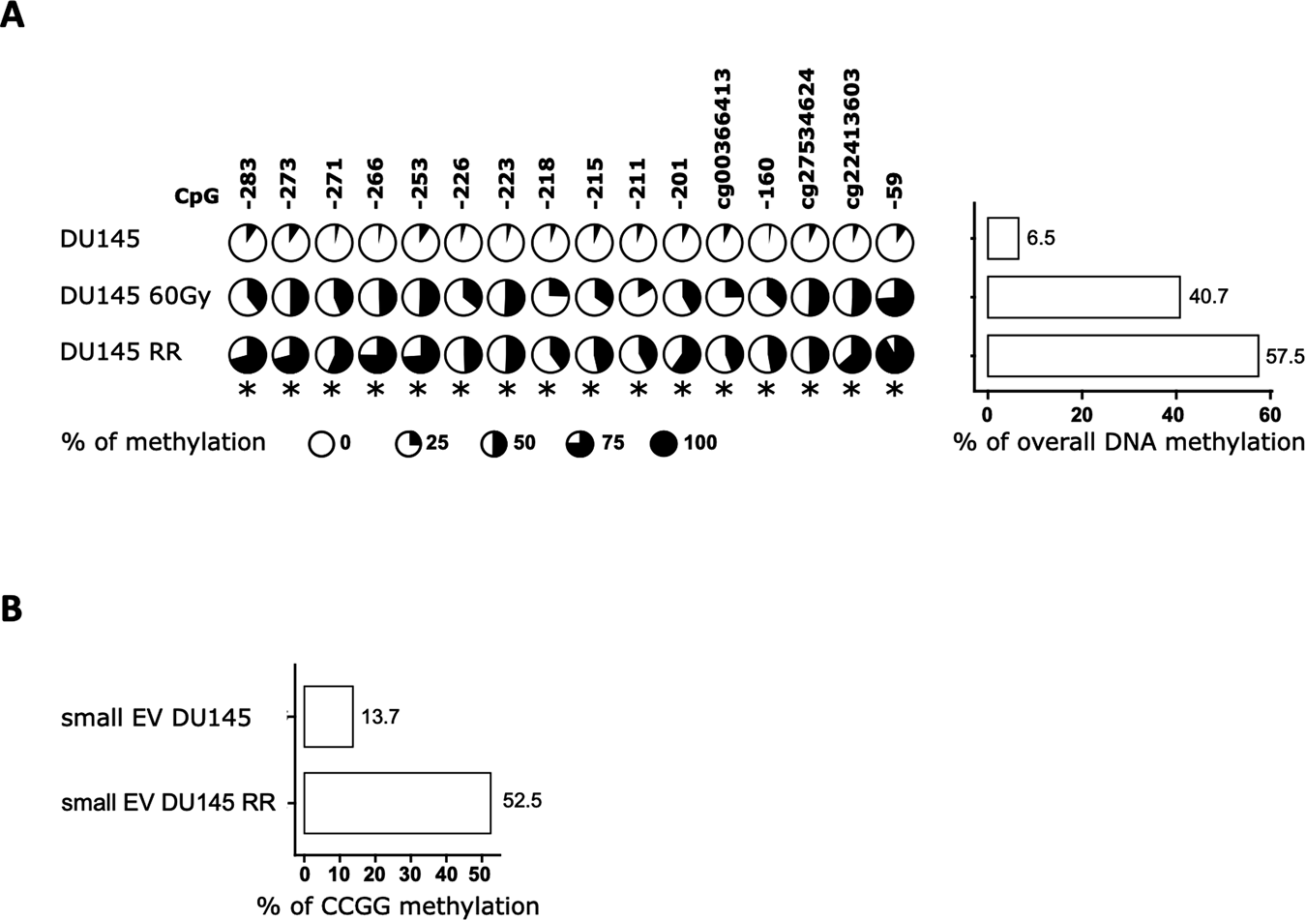
DNA methylation levels of miR-200c-3p promoter reflect its expression. **A** DNA methylation levels of the *MIR200/141* promoter in DU145 parental cells, cells irradiated for a total of 60 Gy undergoing radioresistance acquisition, and radioresistant cells, measured by pyrosequencing. CpG sites are represented according to the beginning of the pre-miR-200c sequence. Kruskal–Wallis: *p < 0.05. *n* = 3. **B** Average levels of DNA methylation on the *MIR200/141* promoter in the small EVs derived from DU145 RR and parental cells measured by methyl-qPCR.

We then studied the DNA methylation status of the *MIR200C* promoter to determine whether its downregulation was due to a gain in methylation. We measured the DNA methylation level of 16 CpGs ranging from the TSS (Transcriptional Start Site) to −300 bp, in DU145 and DU145 RR cells, but also in DU145 cells during the process of acquiring resistance. DU145 60 Gy cells received a cumulative dose of 60 Gy of irradiation but were not significantly more resistant to irradiation than native DU145 cells, unlike DU145 RR cells which received a cumulative dose of 90 Gy of radiation (Supp Fig. 1B). By pyrosequencing, we found that the *MIR200C* promoter is slightly methylated in DU145 cells, with an average promoter-wide methylation level of 6.5%, whereas it is highly methylated in the DU145 RR cells, ranging from 45% in CpG -211 to nearly 100% in CpG -59, with a promoter-wide average of 57.5% (Fig. 2A). Interestingly, we observed an overall promoter methylation level of 40.7% in DU145 cells acquiring radiation resistance (Fig. 2A).

Circulating tumor DNA is used to evaluate tumor-specific mutations for minimal residual disease and response to treatment monitoring but epigenetic features of circulating DNA, such as methylation patterns, have also been recently investigated. We, therefore, measured DNA methylation at the *MIR200C* promoter in DNA extracted from small EVs of DU145 RR and parental cells using the methyl-qPCR method, which is more suitable for small quantities of DNA than bisulfite-pyrosequencing. We confirmed that methyl-qPCR could discriminate between methylated and unmethylated *MIR200C* promoters and that methylation of *MIR200C* promoter on extracellular DNA reflects its methylation level in the cell (Fig. 2B).

Overall, the DNA methylation level at the *MIR200C* promoter in extracellular DNA could be another interesting biomarker of RR in PCa.

### Exogenous miR-200c-3p resensitizes resistant cells to radiation by impeding DNA repair

Since miR-200c-3p expression is repressed in RR cells, we wondered whether this miRNA was merely a biomarker or whether it played a role in the acquisition of resistance. We therefore investigated whether RR cells could be resensitized to irradiation by adding it exogenously.

**Fig. 3:**
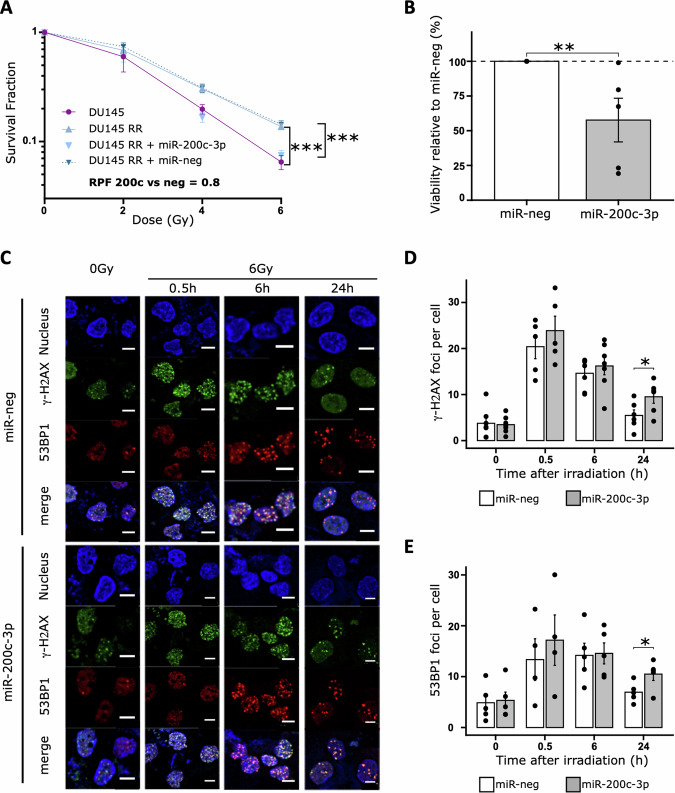
miR-200c-3p decreases radiation-resistant cell survival by delaying DNA repair. **A** Radiation clonogenic survival assays were performed on radioresistant DU145 (DU145 RR) cells transiently transfected with control (miR-neg) or miR-200c-3p mimic. Means and SEM are represented. Wilcoxon test one side at 6 Gy DU145 RR vs DU145+miR-200c-3p or vs DU145: ***p < 0.001, *n* > 4, RPF radiation protection factor. **B** Viability test of DU145 radioresistant cells transfected with control or miR-200c-3p mimic. Twenty-four hours after the transfection, the cells were irradiated with 6 Gy before measuring the viability of the cells 24 hours later with a Cell-Titer Glo kit. The luminescence was expressed relative to that in the miR-neg condition. Wilcoxon test one side, **p < 0.01, *n* = 5. **C** Representative images of confocal microscopy images for the detection of γ-H2AX foci and 53BP1 foci. Nuclei were stained with Hoechst (blue). In green, the phosphorylation of S139 (γ-H2AX) of histone H2AX, in red 53BP1 protein, in DU145 RR cells transfected with miR-neg or miR-200c-3p, at 30 min, 6 h or 24 h after irradiation at 6 Gy. White bar = 10 μm. **D** Plot of the number of γ-H2AX foci number per cell. For each condition, more than 50 cells were analyzed per replicat, *n* = [5–7], Wilcoxon test one side, *p < 0.05. **E** Plot of the number of 53BP1 foci number per cell. For each condition, more than 50 cells were analyzed per replicat, *n* = [4–5], Wilcoxon test one side, *p < 0.05.

Indeed, DU145 RR cells transfected with miR-200c-3p had significantly lower radiation resistance upon irradiation with 6 Gy than DU145 RR cells transfected with control miRNA (miR-neg) (*p* < 0.001) (RPF _miR-200c-3P vs miR-neg_ = 0.8) (Fig. 3A, Supp Fig. 2). Exogenous miR-200c-3p also decreased the viability of the cells, 24 hours after irradiation (6 Gy) (Fig. 2B), suggesting that this miRNA promotes the death of radioresistant cells.

To further investigate the mechanisms by which miR-200c-3p promotes DU145 RR cell radiosensitivity, we measured the γ-H2AX expression, a marker of radiation-induced DSBs, by immunofluorescence. Up to 6 h after 6 Gy irradiation, the number of γ-H2AX foci remained very close between the DU145 RR cells transfected with miR-200c-3p and those transfected with control miRNA. By 24 h however, there was a significantly greater number of γ-H2AX foci in the DU145 RR cells transfected with miR-200c-3p than in those transfected with the control miRNA (Fig. 3C, D). Consistent with these findings, we observed a greater level of 53BP1 (tumor suppressor p53 binding protein 1), a DDR mediator that is recruited to DNA DSBs and induces NHEJ repair, in DU145 RR cells transfected with miR-200c-3p than in those transfected with control miRNA (Fig. 3C, E).

Together, our results suggest that miR-200c-3p decreases DNA repair after irradiation and may, by this way, resensitize radioresistant cells.

### miR-200c-3p impairs DNA repair through HP1α downregulation

To identify targets of miR-200c-3p that are involved in DNA repair, we conducted 3′ RNA sequencing of DU145, DU145 RR, and DU145 RR cells transfected with miR-200c-3p or with a control miRNA.

**Fig. 4:**
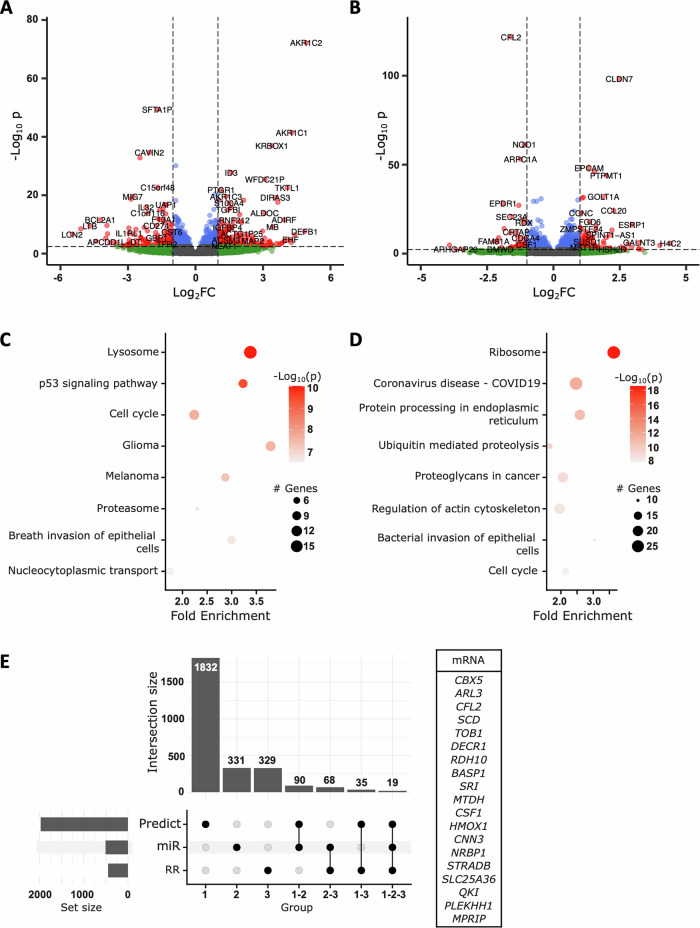
Radiation-resistant and radiation-sensitive PCa cells have distinct transcriptomes. **A** Volcano plot of differentially expressed mRNAs in DU145 RR cells compared to DU145 cells. The red dots on the left represent significantly downregulated mRNAs in DU145 RR cells, and those on the right represent upregulated mRNAs compared to those in DU145 cells. In green, mRNAs with a log2FC > 0.5 or <-0.5 are shown. In red, mRNAs with an adjusted *p* value < 0.05. **B** Volcano plot of differentially expressed mRNAs in DU145 RR cells transfected with miR-200c-3p versus those transfected with a control miRNA. The red dots on the left represent significantly downregulated mRNAs in DU145 RR cells transfected with miR-200c-3p, and those on the right represent upregulated mRNAs compared to those in cells transfected with control miRNA. In green, mRNAs with a log2FC > 0.5 or <−0.5 are shown. In red, mRNAs with an adjusted *p* value < 0.05. **C** Top8 of KEGG pathway enrichment of the DU145 RR-deregulated mRNAs **D** Top8 of KEGG pathway enrichment of the miR-200c-3p transfected DU145 RR-deregulated mRNAs. **E** Upset plot showing intersections between mRNAs predicted to be miR-200c-3p targets through algorithms (predict), mRNAs upregulated in radioresistant cells (RR), and mRNAs downregulated in DU145 RR cells transfected with miR-200c-3p (miR). Right, list of the 19 mRNAs common to all three groups.

We found 934 significantly differentially expressed mRNAs between DU145 RR cells and parental cells (Fig. 4A, Supp Table 3), among which 482 were downregulated in DU145 RR cells, and 452 were upregulated. By analyzing the transcriptomes of DU145 RR cells transfected with miR-200c-3p and control miRNA, we identified 1114 differentially expressed mRNAs (Fig. 4B, Supp Table 4), among which 606 were upregulated in miR-200c-3p transfected cells and 508 were downregulated. KEGG pathway analysis showed enrichment of various functions involved in proliferation, such as the cell cycle pathway (*p* value = 2.19e^−08^ in the DU145 RR cells compared with the DU145 cells and *p* value = 1.59e^-08^ in the DU145 RR cells with miR-200c-3p compared with the control miRNA) (Fig. 4C, D), as well as in DNA repair, such as the NHEJ pathway (*p* value = 0.031 and 0.024 respectively) (Supp Tables 5 and 6). We then crossed our two transcriptome analyses to identify mRNAs that were upregulated in DU145 RR cells compared to DU145 cells; and downregulated in cells transfected with miR-200c-3p (Supp Table 7). We restricted the list to functionally validated target genes of miR-200c-3p available in public databases. In this way, we identified 19 mRNAs as potential miR-200c-3p target genes upregulated in DU145 RR cells (Fig. 4E). One interesting candidate was *CBX5* (chromobox homolog 5), which encodes the HP1α (Heterochromatin Protein 1 alpha) protein, which is involved in heterochromatin formation and closely associated with DNA repair mechanisms [20–22].

**Fig. 5:**
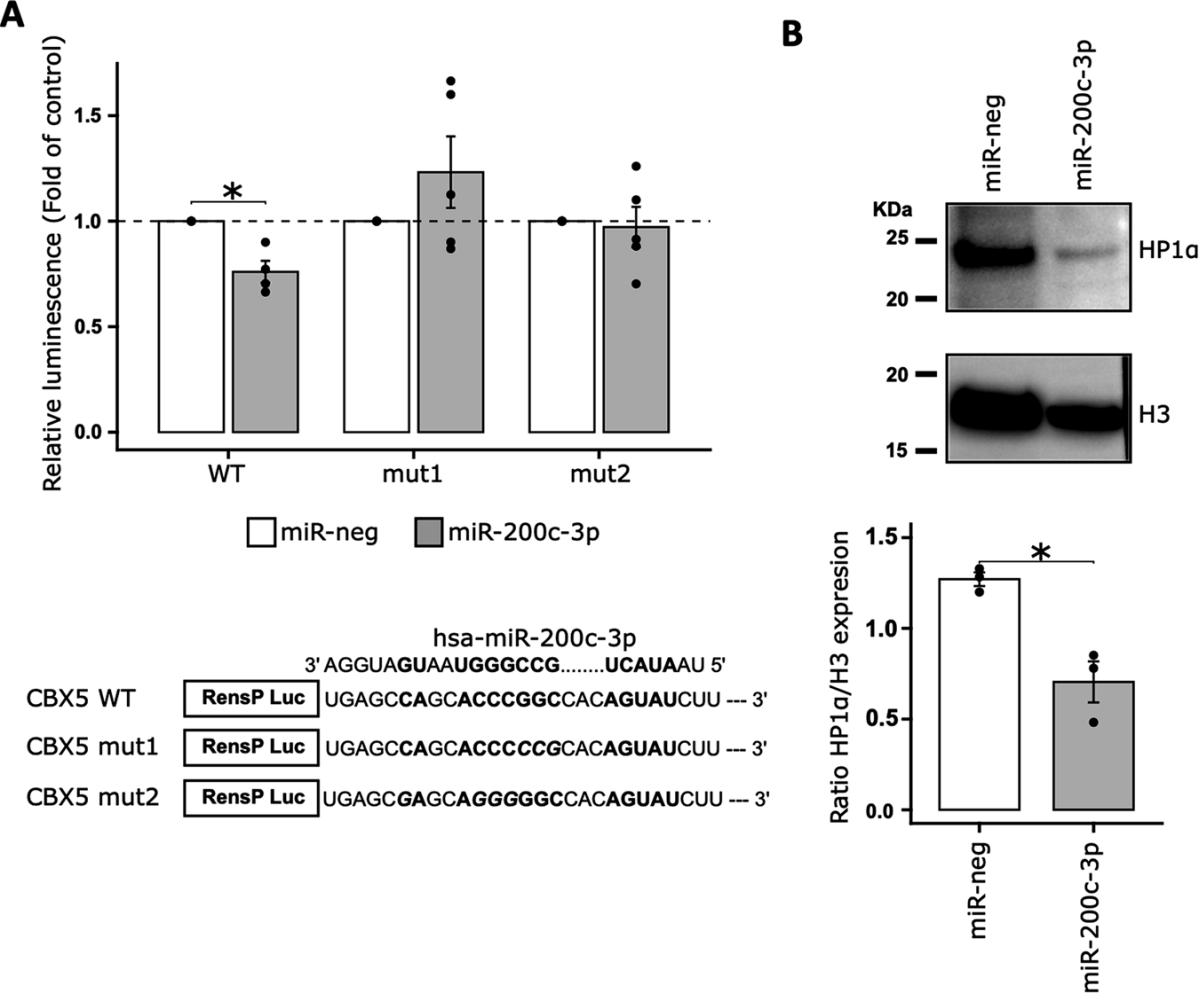
miR-200c-3p directly targets *CBX5 mRNA.* **A** Relative expression of luminescence of HEK-293 cells transfected with 66 nM of miR-200c-3p (grey) or control miRNA (miR-neg) (white) and WT or mutated constructs in the miR-200c-3p binding site (20 ng). Luminescence expression is relative to the luminescence of miR-neg transfected cells. *n* = [4–5], Wilcoxon test one side, *p < 0.05. A schematic representation of the wild-type and mutated constructs in the miR-200c-3p-binding site is shown. WT, wild type. **B** Quantification of HP1α expression in DU145 RR cells transfected with miR-200c-3p or control miRNA (miR-neg) by western blot analysis. The optical density of each sample was measured and normalized using a nucleus housekeeping protein (H3) run on the same gel using ImageJ. The data are expressed as relative expression (HP1α/H3). *n* = 3, Wilcoxon test, *p < 0.05.

After validating the 3′ RNA sequencing results for *CBX5* by RT-qPCR (Supp Fig. 3), we evaluated the direct targeting of *CBX5* by miR-200c-3p. To this end, we transiently co-transfected miR-200c-3p into HEK-293 cells with selected *CBX5* 3′UTR sequences downstream of the RenSP luciferase reporter gene (Fig. 5A). We found that miR-200c-3p binds directly to the *CBX5* 3′UTR as observed by the decrease in luminescence in the presence of the wild-type (WT) sequence. The introduction of 3 (mut1) to 4 (mut2) mismatches abolished this interaction, as shown by the absence of luminescence loss (Fig. 5A).

We also measured the impact of miR-200c-3p transfection on HP1α protein expression by western blot analysis. This confirmed that the downregulation of *CBX5* mRNA by miR-200c-3p leads to a decrease in the expression of its protein, HP1α (Fig. 5B).

Taken together, these data indicate that the impact of miR-200c-3p on the RR is mediated at least in part by the regulation of HP1α expression.

### Silencing of miR-200c-3p in radioresistant cells is a common mechanism

**Fig. 6:**
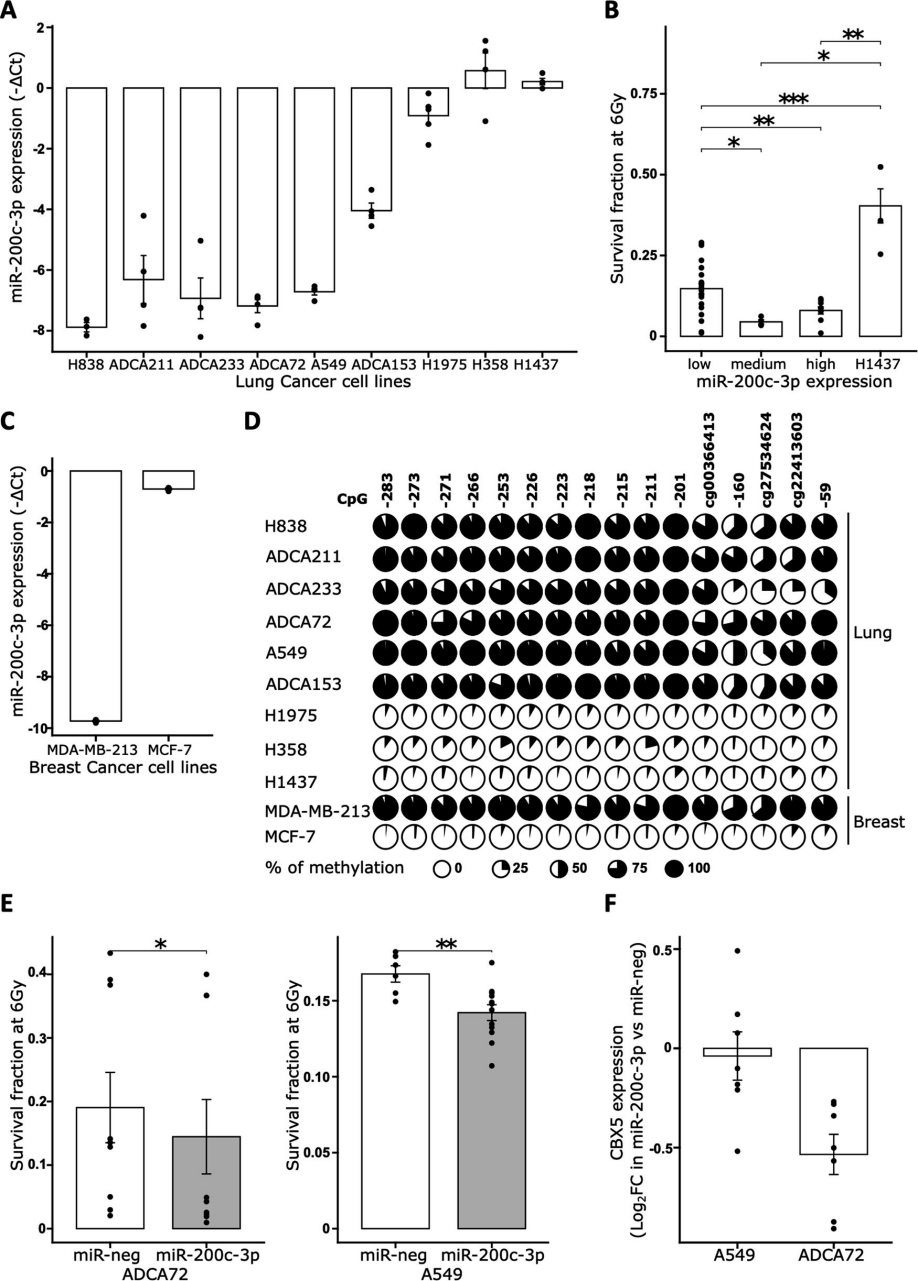
Silencing of miR-200c-3p is associated with radiation resistance in other cancers. **A** miR-200c-3p expression in lung cancer cell lines. The expression of miR-200c-3p is expressed relative to snord44, an endogenous housekeeping RNA. Means and SEM of *n* = 4 experiments per cell line are represented. **B** Relationships between radioresistance at 6 Gy and miR-200c-3p expression in lung cancer cell lines. Lung cancer cell lines were grouped according to their miR-200c-3p expression. Means and SEM of *n* = 3 experiments per cell line are shown. Wilcoxon test. **C** miR-200c-3p expression in breast cancer cell lines. The expression of miR-200C-3p is expressed relative to snord44, an endogenous housekeeping RNA. Means and SEM of *n* = 4 experiments per cell line are represented. **D** DNA methylation levels of the *MIR200/141* promoter in lung and breast cancer cell lines measured by pyrosequencing. CpG sites are represented according to the beginning of the pre-miR-200c sequence. **E** Survival fraction at 6 Gy of A549 and ADCA72 cell lines transfected with miR-200c-3p or a control miRNA. 9 experiments were performed for each cell line. Wilcoxon test. **F** RT-qPCR of CBX5 mRNA in A549 and ADCA72 cell lines transfected with miR-200c-3p compared to a control miRNA. *n* = 7 experiments per cell line. *p < 0.05, **p < 0.01, ***p < 0.001.

Next, we wondered whether this mechanism is specific to PCa by measuring miR-200c-3p expression in cell lines from other cancers treated by radiotherapy. In lung cancer, we found that 5 cell lines (H838, ADCA211, ADCA233, ADCA72 and A549) exhibited a low level of miR-200c-3p expression, 3 cell lines (H1975, H358 and H1437) had a high level of expression and one had an intermediate level of expression (ADCA153) (Fig. 6A). Investigated healthy cells seemed to show a low level of miR-200c-3p expression (Supp Fig. 5). Interestingly, except for H1437 which was not really efficient at growing from a single cell to a colony, we observed a good correlation between miR-200c-3p expression and the cell survival fraction after 6 Gy irradiation (Fig. 6B, Supp Fig. 4A, B). Indeed, cell lines with low expression levels had a significantly greater survival fraction than did those with high expression levels of miR-200c-3p. We also evaluated this correlation in two breast cancer cell lines, one known to be resistant to irradiation, *i.e*. MDA-MB-231, and another one known to be sensitive to RT (MCF-7) [23], and confirmed that miR-200c-3p expression was significantly lower in resistant MDA-MB-231 cells than in MCF-7 cells (Fig. 6C). Moreover, as observed in PCa, miR-200c-3p expression was strongly correlated with the DNA methylation level of its promoter in lung and breast cancer cell lines (Fig. 6D).

We next investigated whether these RR cells expressing low levels of miR-200c-3p could be resensitized to irradiation by adding it exogenously, as observed in PCa cell lines. We selected two lung cancer cell lines that readily form clones and found that ADCA72 and A549 cells transfected with miR-200c-3p had significantly lower radiation resistance upon irradiation with 6 Gy than ADCA72 and A549 cells transfected with control miRNA (miR-neg) (respectively *p* < 0.05 and *p* < 0.01) (Fig. 6E, Supp Fig. 6A). To evaluate whether in these cells *CBX5* was also an important target of miR-200c-3p, we evaluated first its expression by RT-qPCR. We observed a good correlation between miR-200c-3p expression and its potential target *CBX5* in lung cancer cell lines (Supp Fig. 6B). Next, we measured by RT-qPCR its expression in ADCA72 and A549 cells transfected with miR-200c-3p or a control miRNA. In A549 cell line, *CBX5* did not seem the main target of miR-200c-3p, whereas, in ADCA72 cell line, we observed the same decrease in *CBX5* expression when cells were transfected with miR-200c-3p compared to miR-neg, as in DU145 RR cells (Fig. 6F). We believe that the mechanisms involved in resistance between these cell lines are common.

We concluded that silencing of miR-200c-3p is a common mechanism of RR in different types of cancer cells.

## Discussion

The identification of biomarkers and signaling pathways involved is important for the management of PCa RR. In this study, we showed that miR-200c-3p expression is silenced by DNA methylation in resistant tumor cell lines. As miRNA and circulating DNA assays from liquid biopsy samples are promising new biomarkers, we also demonstrated that miR-200c-3p expression can be monitored in small EVs as well as its promoter DNA methylation level. Functionally, miR-200c-3p overexpression resensitizes tumor cells to radiotherapy in association with impairing DDR.

The miR-200 family was previously shown to be involved in therapeutic resistance, mainly related to chemotherapy. The first evidence regarding the association between miR-200c-3p and radiotherapy resistance came from a study on breast cancer cells in which low levels of miR-200c-3p expression were correlated with radiotolerance, and ectopic miR-200c-3p expression enhanced irradiation-induced apoptosis by directly targeting TBK1 (TANK-binding kinase 1) [24]. Since then, several other targets of miR-200c-3p have been shown to regulate RR, including UBQLN1 (ubiquilin 1) [25] and PRDX2 (peroxiredoxin 2) [26]. Our study provides novel insight into the function of miR-200c-3p function by identifying *CBX5* mRNA as a direct target gene. *CBX5* encodes for HP1α, a non-histone chromosomal protein. This protein is known mainly for its involvement in heterochromatin formation through interaction with Suv39h1, and both of these proteins are essential for HR after DSBs [27]. Following ionizing radiation, HP1α accumulates at DSBs through its recruitment by p150CAF-1 [22], the largest subunit of chromatin assembly factor 1 [21]. This leads to rapid reorganization of DSBs and expansion of HP1α domains, and repair foci shift to the periphery of heterochromatin [20]. Several studies have demonstrated that loss of HP1α impairs the recruitment of RAD51 and BRCA1 (breast cancer 1, early onset), two HR proteins, that delay DSB repair after irradiation [21, 28], and lead to radiosensitivity [21, 22]. This finding highlights that HP1α is a critical component for proper DNA damage signaling and repair after irradiation. Moreover, our 3′SRP identified other potential miR-200c-3p target genes that could be involved in radiation resistance, including *CLU* (clusterin), a protein that is overexpressed in radioresistant LNCaP prostate tumor cells [29]. Our sequencing also identified interesting candidates such as *CFL2* (cofilin 2), whose inactivation in nasoparyngeal carcinoma decreased cell proliferation, while apoptosis and radiosensitivity were enhanced [30], or *MTDH* (metadherin) [31–33], *MPRIP* (myosin phosphatase Rho interacting protein) [34] and *TOB1* (Transducer of ERBB2, 1) [35–37] all involved in response to radiation in cancer cells.

In radioresistant PCa, lung, and breast cancer cell lines, we found that miR-200c-3p expression was silenced by DNA methylation. DNA methylation of the CpG island promoter of miRNA genes is a common epigenetic mechanism for silencing tumor suppressor miRNAs in human cancer [38]. Pyrosequencing analysis revealed that ~6.5% of the parental DU145 cells were methylated, whereas more than 50% of the DU145 RR cells were methylated, indicating that the methylated clones were selected under RT due to their selective advantage. However, there have been limited studies of the effect of radiation on DNA methylation. Antwih et al. reported that acute irradiation of MDA-MB-231 breast cancer cells induces DNA methylation changes at several CpGs located near genes involved in the cell cycle, DNA repair, and apoptosis pathways [39]. Previously, Kim et al. identified 1091 differentially methylated regions between a lung cancer cell line resistant (H1299) and one sensitive (H460) to radiation.

Moreover, the determination of methylation in circulating tumor DNA may be an interesting option for the monitoring response to RT [40]. In our study, we demonstrated that the level of extracellular DNA methylation reflects the DNA methylation status of cells on the *MIR200C* promoter, but also the expression of miR-200c-3p, and could therefore be used as a circulating biomarker. Tumor-circulating DNA is increasingly being used as a noninvasive disease-monitoring tool in cancer. The identification of specific methylation signatures can help monitor treatment response, such as the emergence of therapy-resistant tumor cells characterized by a high DNA methylation level on the MIR200C/141 promoter on circulating DNA.

The use of tumor-circulating nucleic acids or those included in small EVs as biomarkers seems to depend on the type and the stage of cancer. Indeed, as their origins are different, the former coming from apoptotic and necrotic cells and the latter from living cells through an active process, the information they convey are sometimes distinct. Karacam et al. showed that both circulating DNA and EVs could be used as diagnostic biomarkers in glioma patients [41], while Nasu et al. demonstrated that RNAs from EVs gave more robust and consistent findings in plasma samples than circulating RNA as they mitigated the impact of batch effects derived from hemolysis [42]. Similarly, Wan et al. found that EV-derived DNA was superior to circulating DNA for mutation detection in early-stage non-small cell lung cancer (NSCLC), while the advantages disappeared in advanced-stage NSCLC [43]. Here, we demonstrated that both can be used as biomarkers of resistance to RT.

In conclusion, we identified 4 miRNAs that are specific for RR in PCa cell lines, of these, miR-200c-3p was the most relevant candidate since it can be investigated as well, outside cells, in small EVs. Our study highlights the ability of miR-200c-3p to resensitize radioresistant cancer cells to irradiation, and its potential as a biomarker of response to radiotherapy.

## Supplementary information

Supp figure titles

Supp figures and western blots

Supp Tables 1–7

## Data Availability

The data generated in this study are publicly available in the Gene Expression Omnibus (GEO) at GSE246676.
