# Supplementary material for: Loss of miR-200c-3p promotes resistance to radiation therapy via the DNA repair pathway in prostate cancer
Source: Cell Death Dis. 2024 Oct 16;15(10):751. doi: 10.1038/s41419-024-07133-3 (PMC11484813; doi:10.1038/s41419-024-07133-3)
Supplement: Supplementary file 1 — Supp figure titles [file 41419_2024_7133_MOESM1_ESM.docx]

**SUPP FIGURE TITLES**

**Supp Fig 1. A)** Representative photographs of radiation clonogenic survival assays for DU145 and PC3 cells. 500, 500, 2500 and 5000 cells were plated per well for 0Gy, 2Gy, 4Gy and 6Gy condition respectively. Cells were then treated with ionizing radiation. **B)** Radiosensitivity of established radiation resistant DU145 (DU145 RR), DU145 cell lines acquiring radioresistance (DU145 60 Gy) and parental cells (DU145). Growth curves in colony formation assay. Means and SEM are represented. Wilcoxon test, one side Parental vs 90Gy: *: p<0.05, **: p<0.01 for DU145 RR vs DU145 condition. n=6 independent experiments.

**Supp Fig 2. Representative photographs of radiation clonogenic survival assays to test miR-200c-3p effect**. DU145 radioresistant cells (DU145 RR) were transiently transfected with miR-200c-3p or miR-neg mimic, 66nM final. Then, 500, 500, 2500 and 5000 cells were plated per well for 0Gy, 2Gy, 4Gy and 6Gy condition respectively. Cells were then treated with ionizing radiation.

**Supp Fig 3. RT-qPCR of *CBX5* mRNA in DU145 RR versus DU145 cells and in DU145 RR transfected with miR-200c-3p compared to miR-neg.** n= 5-6.

**Supp Fig 4. Modulation of miR-200c-3p expression by DNA methylation and radioresistant phenotype association in NSCLC. A)** Representative photographs of radiation clonogenic assay on A549 and H1975, two lung cancer cell lines. 200 cells were plated per well for each condition. **B)** Radiation clonogenic survival assays in NSCLC according to their methylation status of *MIR200C/141* promoter. In blue: highly methylated, in green: moderately methylated, in purple: weakly methylated. n>4 per cell line.

**Supp Fig 5. miR-200c-3p expression in healthy cells. Expression of miR-200c-3p is expressed relative to snord44, an endogenous housekeeping RNA.** Immune cells are CD8+ T cells freshly sorted from PBMCs, endothelial cells are HUVECs freshly sorted from human umbilical cords and melanocytes are PCS-200-013. Means and SEM of n=4 experiments per cell types are represented.

**Supp Fig 6. miR-200c-3p expression is associated with radiation resistance and *CBX5* expression in lung cancer cell lines. A)** Relationships between radioresistance at 6Gy and the doses of transfected miR-200c-3p in A549 cells. 3 experiments were performed. **B)** Expression of miR-200c-3p expressed relative to snord44, an endogenous housekeeping RNA. Means and SEM of 3 experiments per cell lines.
