# Supplementary figures and images for: Loss of miR-200c-3p promotes resistance to radiation therapy via the DNA repair pathway in prostate cancer
Source: Cell Death Dis. 2024 Oct 16;15(10):751. doi: 10.1038/s41419-024-07133-3 (PMC11484813; doi:10.1038/s41419-024-07133-3)

Supp Fig 1.

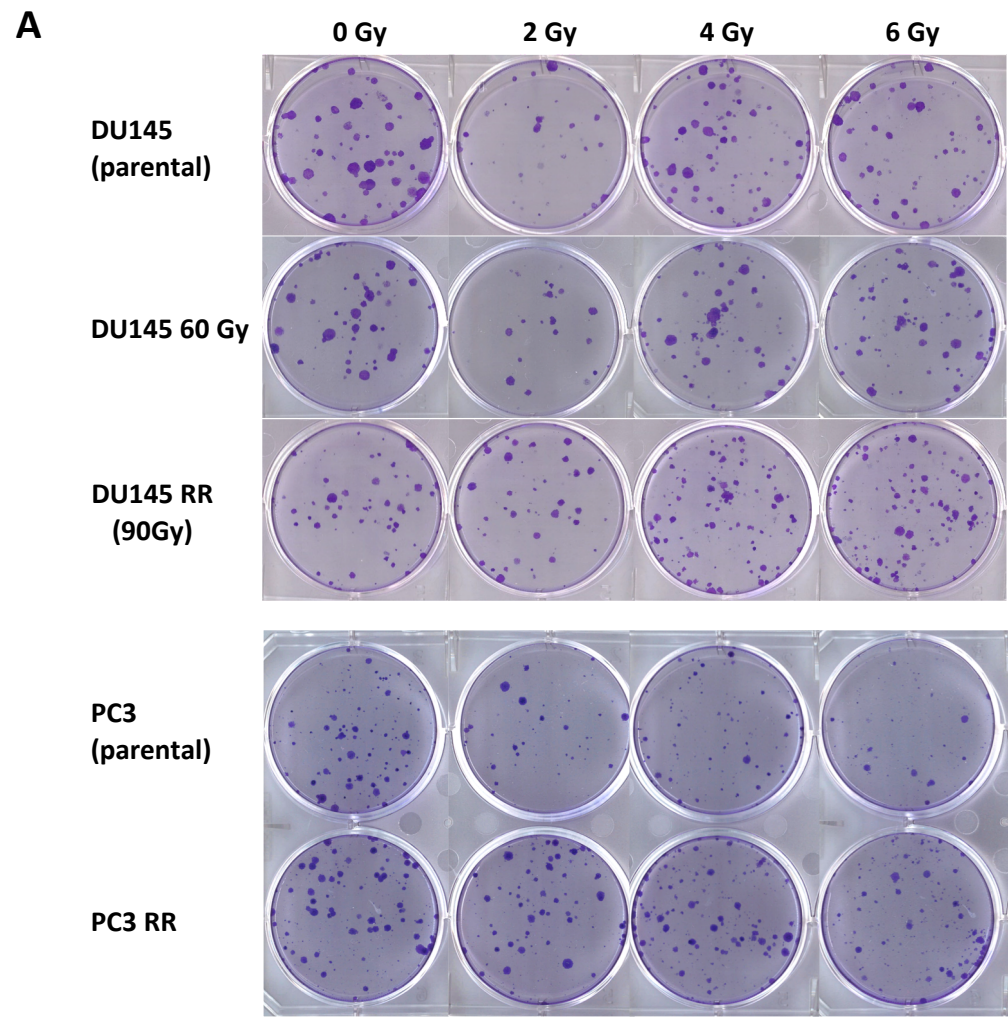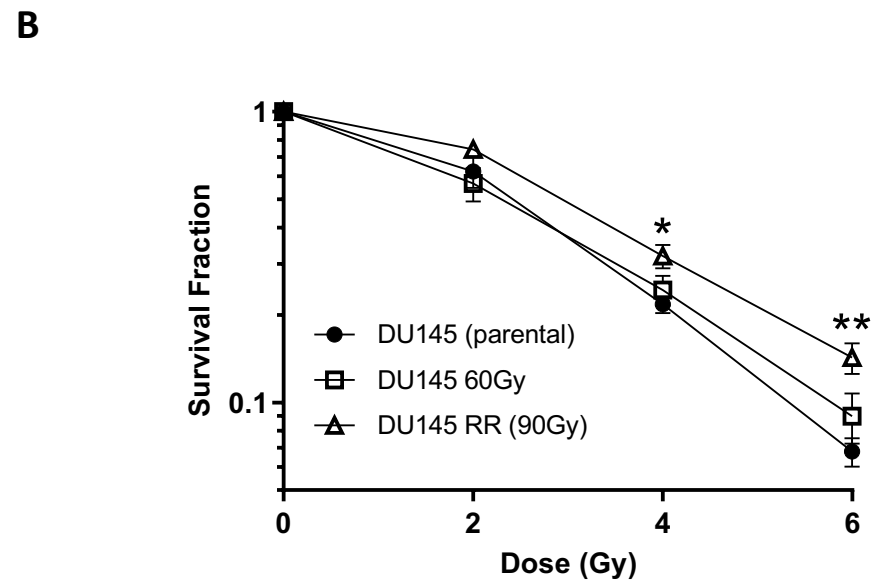

Supp Fig 2.

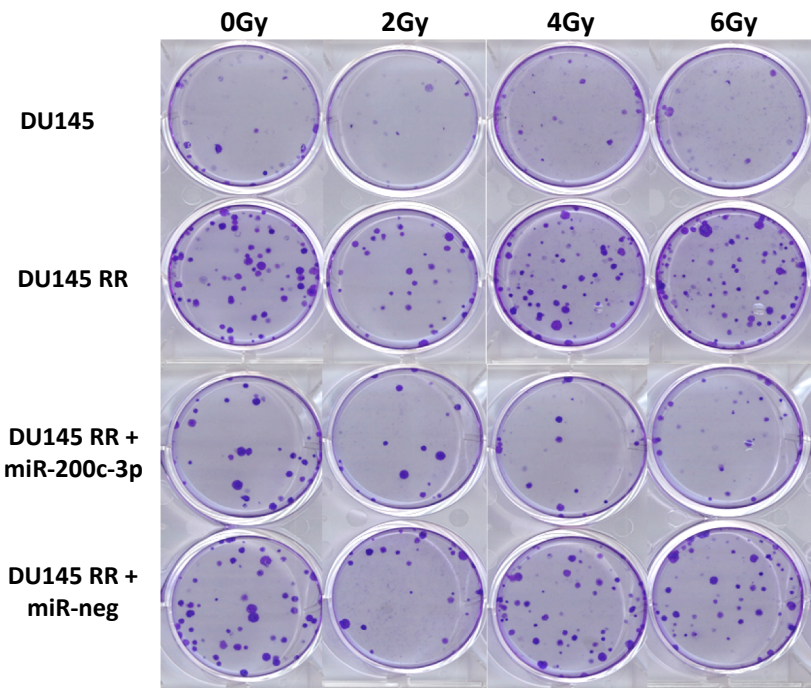

Supp Fig 3.

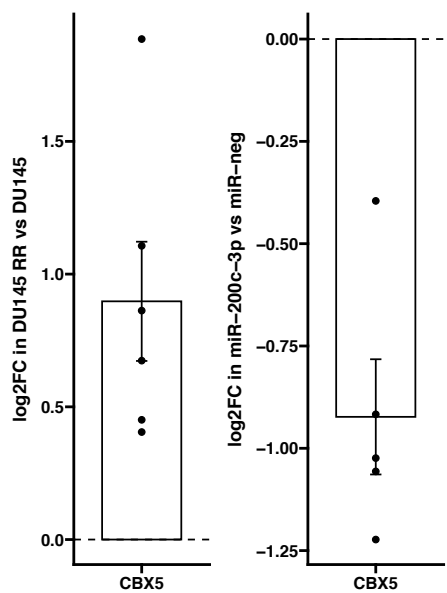

Supp Fig 4.

A

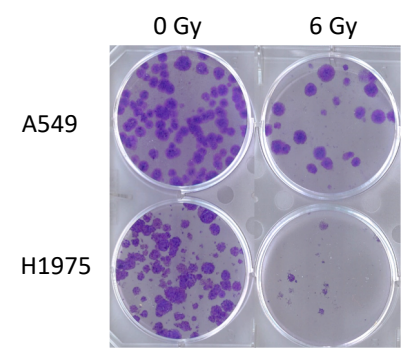

B

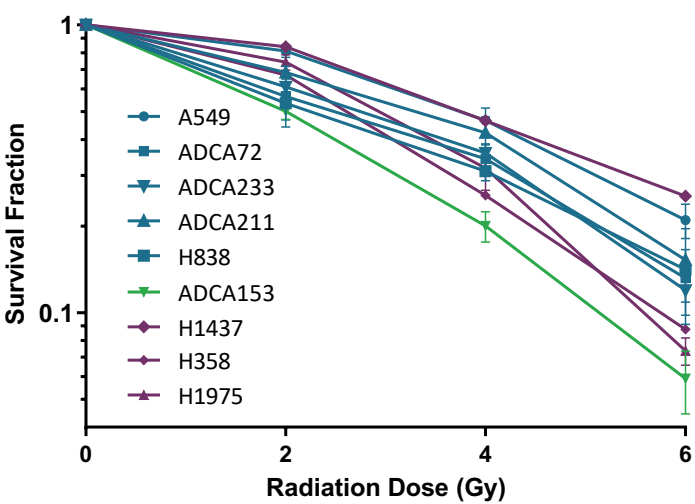

Supp Fig 5.

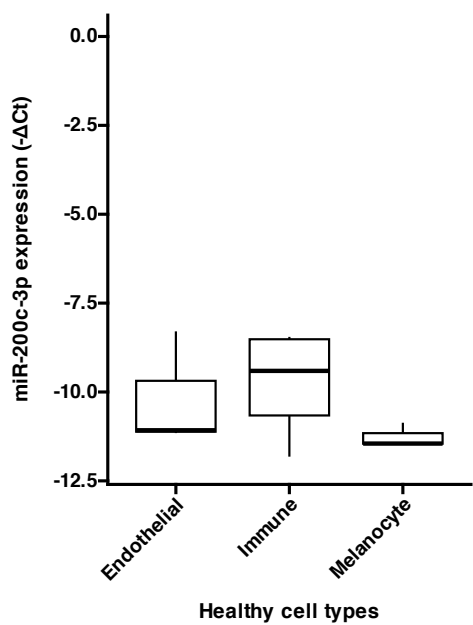

Supp Fig 6.

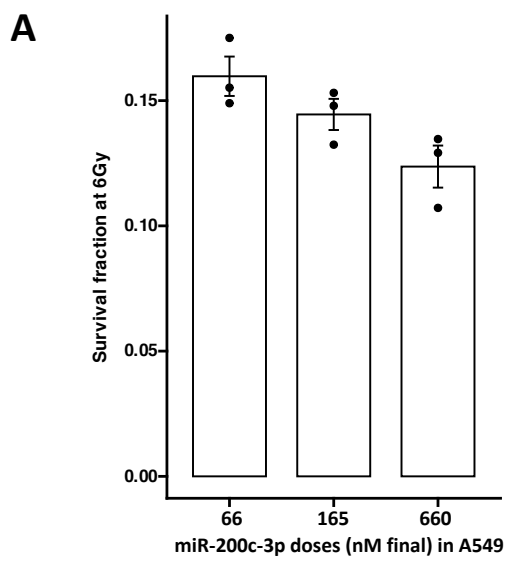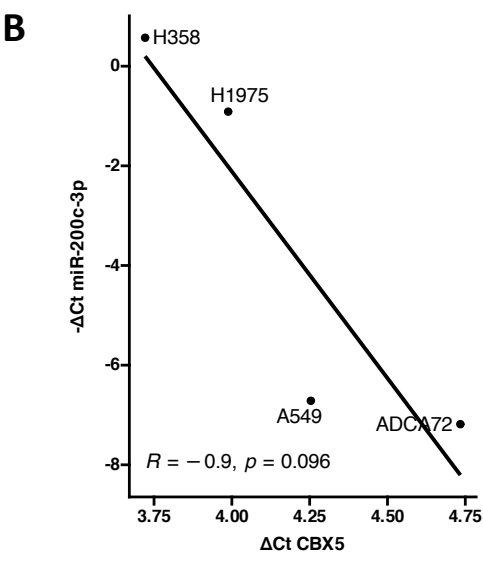

Supp Fig. Original western blot

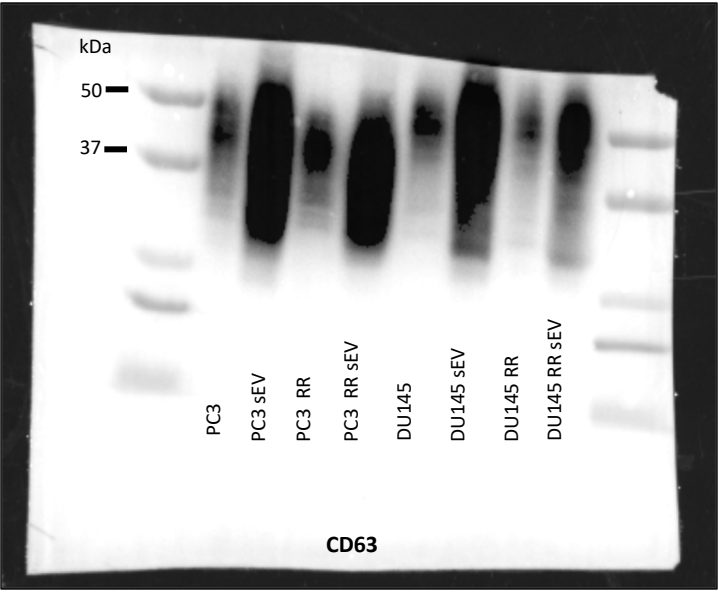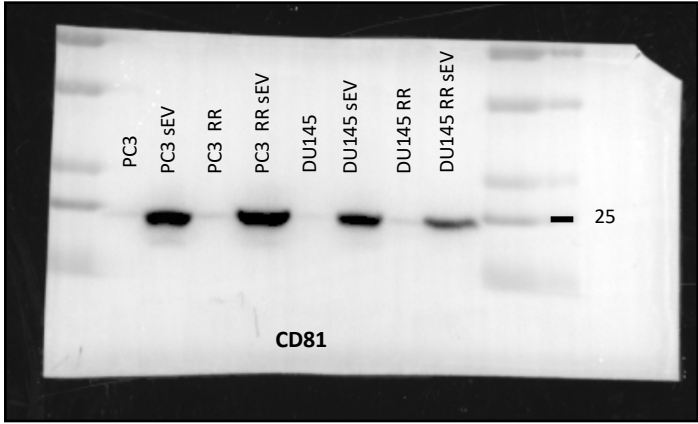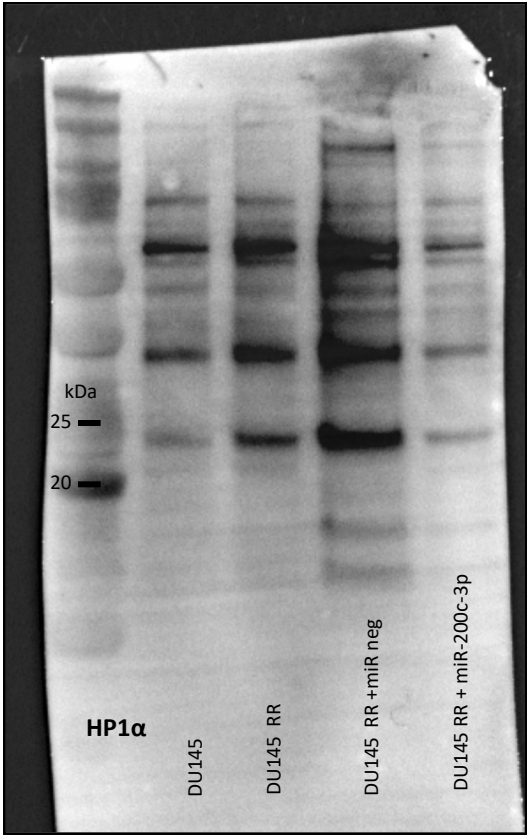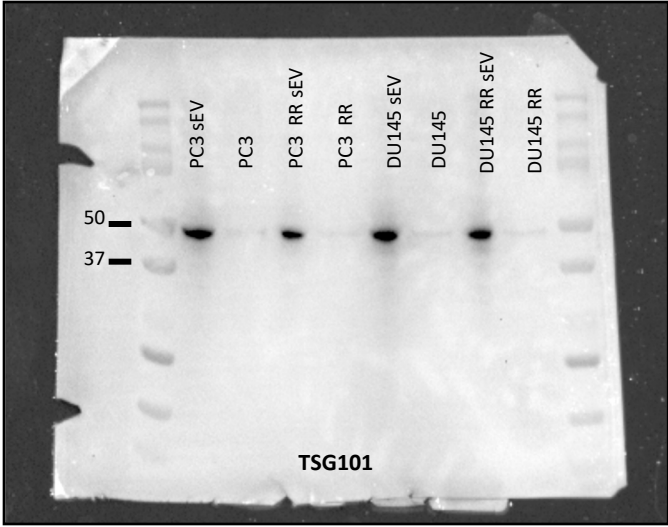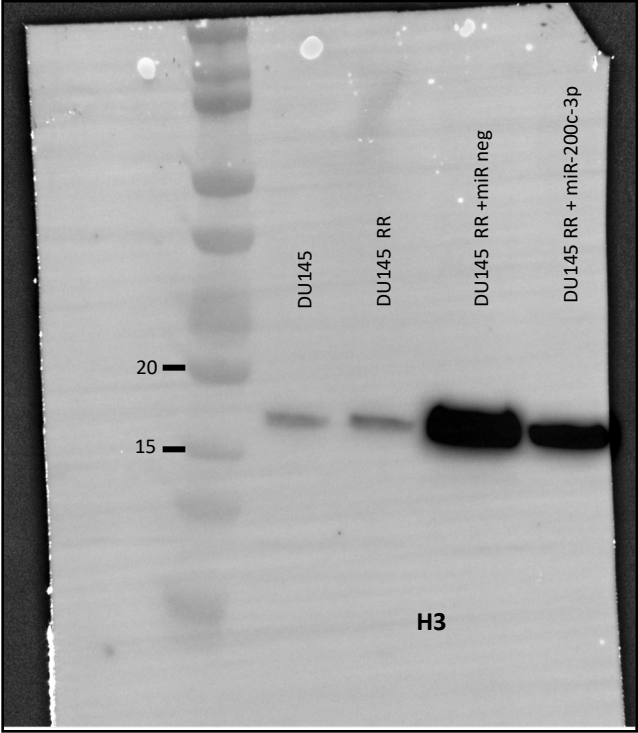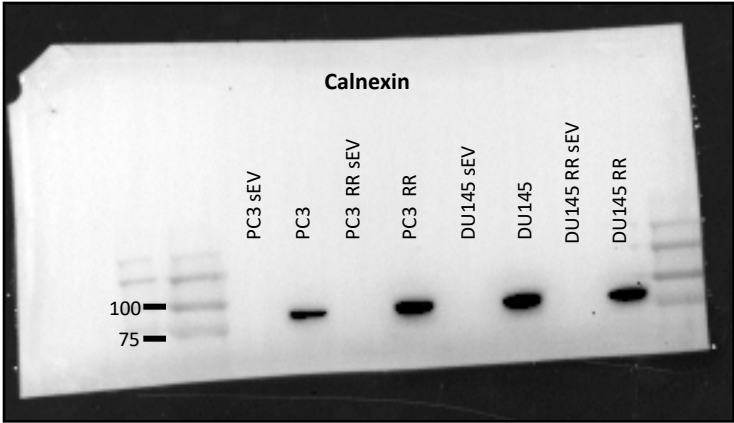

Supplement: Supplementary file 2 — Supp figures and western blots [file 41419_2024_7133_MOESM2_ESM.pdf]
